# The Corner-Stone of Rush Medical College

**Published:** 1867-07

**Authors:** 


### *The Corner-Stone of Rush Medical College*

Was laid with Masonic ceremonial, in due and ancient form. Particulars in next JOURNAL.

